# Reduced stress perfusion in myocardial infarction with nonobstructive coronary arteries

**DOI:** 10.1038/s41598-023-49223-w

**Published:** 2023-12-13

**Authors:** Rebecka Steffen Johansson, Per Tornvall, Peder Sörensson, Jannike Nickander

**Affiliations:** 1 Department of Clinical Physiology, Karolinska Institutet, Stockholm, Sweden; 2 Klinisk Fysiologi A8:01, Karolinska University Hospital, Solna, Eugeniavägen 23, 171 76 Stockholm, Sweden; 3 Department of Clinical Science and Education, Södersjukhuset, Karolinska Institutet, Stockholm, Sweden; 4 Cardiology Unit, Södersjukhuset, Stockholm, Sweden; 5 Department of Medicine Solna, Karolinska Institutet, Stockholm, Sweden; 6 Department of Cardiology, Karolinska University Hospital, Stockholm, Sweden

**Keywords:** Cardiology, Cardiovascular biology

## Abstract

Myocardial infarction with nonobstructive coronary arteries (MINOCA) has several possible underlying causes, including coronary microvascular dysfunction (CMD). Early cardiovascular magnetic resonance imaging (CMR) is recommended, however cannot provide a diagnosis in 25% of cases. Quantitative stress CMR perfusion mapping can identify CMD, however it is unknown if CMD is present during long-term follow-up of MINOCA patients. Therefore, this study aimed to evaluate presence of CMD during long-term follow-up in MINOCA patients with an initial normal CMR scan. MINOCA patients from the second Stockholm myocardial infarction with normal coronaries study (SMINC-2), with a normal CMR scan at median 3 days after hospitalization were investigated with comprehensive CMR including stress perfusion mapping a median of 5 years after the index event, together with age- and sex-matched volunteers without symptomatic ischemic heart disease. Cardiovascular risk factors, medication and symptoms of myocardial ischemia measured by the Seattle Angina Questionnaire 7 (SAQ-7), were registered. In total, 15 patients with MINOCA and an initial normal CMR scan (59 ± 7 years old, 60% female), and 15 age- and sex-matched volunteers, underwent CMR. Patients with MINOCA and an initial normal CMR scan had lower global stress perfusion compared to volunteers (2.83 ± 1.8 vs 3.53 ± 0.7 ml/min/g, *p* = 0.02). There were no differences in other CMR parameters, hemodynamic parameters, or cardiovascular risk factors, except for more frequent use of statins in the MINOCA patient group compared to volunteers. In conclusion, global stress perfusion is lower in MINOCA patients during follow-up, compared to age- and sex-matched volunteers, suggesting that CMD may be a possible pathophysiological mechanism in MINOCA.

**Clinical Trial Registration: **Clinicaltrials.gov identifier NCT02318498. Registered 2014-12-17.

## Introduction

Myocardial infarction with nonobstructive coronary arteries (MINOCA) is a myocardial infarction (MI) without coronary artery stenosis ≥ 50% on coronary angiography (CAG)^1^ and without a known specific diagnosis causing the acute presentation^2,3^. MINOCA has several possible underlying causes, including coronary microvascular dysfunction (CMD)^4,5^. CMD encompasses functional and structural abnormalities in the coronary microvasculature, leading to insufficient increase in myocardial perfusion in response to increasing oxygen demands, causing myocardial ischemia^6^. Early cardiovascular magnetic resonance imaging (CMR) is recommended for differential diagnosis following MINOCA presentation^3,7–10^, and provides prognostic information^11^. However, standard clinical CMR is normal in 25% of MINOCA cases and thus fails to provide a diagnosis enabling specific treatment^12,13^. New fully quantitative stress CMR perfusion mapping adds the possibility to assess regional and global myocardial perfusion (ml/min/g)^14,15^. Quantitative stress perfusion mapping can detect CMD as reduced global stress perfusion and/or myocardial perfusion reserve (MPR)^16^ and can differentiate CMD from obstructive coronary artery disease (CAD)^17^. CMD has been shown in acute MINOCA and is associated with poor outcome both in the acute stage and long-term^18^. However, long-term presence of CMD following acute MINOCA presentation has to the best of our knowledge not been examined previously. Moreover, multiparametric CMR including quantitative stress perfusion has not been utilized in the evaluation of MINOCA patients, despite its ability to non-invasively and without radiation assess cardiac function, structure and tissue properties. Therefore, we aimed to evaluate presence of CMD in MINOCA patients with an initial normal CMR scan in long-term follow-up, using comprehensive multiparametric CMR, including quantitative stress perfusion mapping. The hypothesis was that MINOCA patients would have lower global stress perfusion compared to sex- and age-matched volunteers with similar comorbidities but without symptomatic ischemic heart disease (IHD), indicating CMD.

## Methods

### Study group

MINOCA patients were identified from the second Stockholm Myocardial Infarction with Normal Coronaries (SMINC-2) study, which was a prospective, non-randomized study of MINOCA patients performed during 2014–2018 in five hospitals in Stockholm, Sweden. SMINC-2 utilized early CMR including cine imaging, native T1 and extracellular volume (ECV) mapping and late gadolinium enhancement (LGE) imaging for differential diagnosis of MINOCA^13^. Inclusion criteria included fulfillment of the diagnostic criteria of MI^1^ and nonobstructive coronary arteries (stenosis < 50%) on CAG, determined visually. Exclusion criteria included lack of sinus rhythm on admission electrocardiogram (ECG), pulmonary embolism, previous MI, known cardiomyopathy, severe asthma or chronic obstructive pulmonary disease, decreased kidney function (serum creatinine of > 150 μmol/l), pacemaker, or claustrophobia. All patients were invited to follow-up CMR 6 months after index event^19^, the last follow-up scan was performed in September 2019.

**Figure 1:**
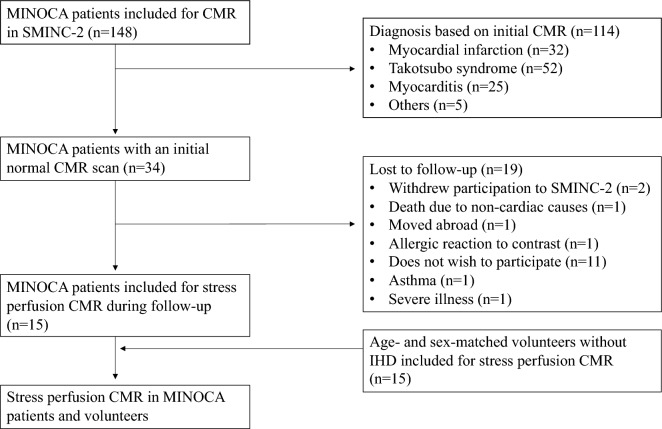
Flow chart of study cohort. The figure shows inclusion and exclusion of MINOCA patients and volunteers into the study. *SMINC-2* Stockholm myocardial infarction with normal coronaries-2, *MINOCA* myocardial infarction with nonobstructive coronary arteries, *CMR* cardiovascular magnetic resonance imaging, *IHD* ischemic heart disease.

Patients were invited to participate in the present study if their initial CMR scan and 6 months follow-up scan were read as normal by two independent level 3 CMR physicians. Furthermore, sex- and age-matched volunteers without IHD history were recruited by advertising at Karolinska Institutet (Fig. 1). Stress perfusion CMR was performed between March 2020 and February 2022. Clinical data including biochemical cardiac markers at index event, as well as presence of cardiovascular risk factors and medications at follow-up, were obtained by interviews or from medical records. The Seattle Angina Questionnaire 7 (SAQ-7) was used to quantify symptoms of myocardial ischemia, and results were summarized as the SAQ-7 summary score^20^. Ethical approval has been granted for all procedures in the study by the Swedish Ethical Review Authority (Dnr 2014/131-31/1, 2017/2415-32/1, 2021-06837-02), all study procedures were carried out in accordance with relevant guidelines and regulations as per the Declaration of Helsinki for involving human participants, and all participants provided written informed consent.

### Image acquisition

CMR was performed supine with a Magnetom Aera® 1.5 Tesla (T) scanner (Siemens Healthcare, Erlangen, Germany), with a phased-array 18-channel body matrix coil and a spine matrix coil. A venous blood sample was drawn to determine hematocrit and blood creatinine prior to imaging. Full coverage retrospective electrocardiographic (ECG)-gated balanced steady state free precession (bSSFP) cine imaging was acquired in standard three long-axis and short-axis slices. Typical imaging parameters were flip angle (FA) 68°, pixel size 1.4 × 1.9 mm^2^, slice thickness 8.0 mm, echo time (TE)/repetition time (TR) 1.19/37.05 ms, matrix size 256 × 144 and field of view (FOV) 360 × 270 mm^2^.

Using first pass perfusion imaging, quantitative perfusion maps (ml/min/g) were acquired in three short-axis slices (basal, midventricular, apical) during administration of an intravenous contrast agent bolus (0.05 mmol/kg, gadobutrol, Gadovist, Bayer AB, Solna, Sweden), during adenosine infusion (140 µg/kg/min or increased according to clinical routine to 210 µg/kg/min in the absence of adequate response to adenosine (Adenosin, Life Medical AB, Stockholm)) and in subsequent rest. Adenosine response was assessed clinically based on symptoms and heart rate response and was evaluated by the rate pressure product (RPP) in rest and stress. Adenosine and contrast agent were administered in two different cannulas. Subjects abstained from caffeine for 24 h and nicotine for 12 h prior to CMR examination. A distributed tissue exchange model was used to compute the perfusion maps^21^ and the Gadgetron inline perfusion mapping software was used to generate the perfusion maps^14^.Typical imaging parameters were bSSFP single shot readout, FA 50°, slice thickness 8.0 mm, TE/TR 1.04/2.5 ms, bandwidth 1085 Hz/pixel, FOV 360 × 270 mm^2^ and saturation delay/trigger delay (TD) 95/40 ms.

Using an ECG-gated Modified Look-Locker inversion recovery (MOLLI) 5s(3s)3s prototype sequence, native T1 maps were acquired in three short-axis slices. Typical imaging parameters were single shot SSFP in end-diastole, FA 35°, pixel size 1.4 × 1.9 mm^2^, slice thickness 8.0 mm, imaging duration 167 ms, TE/TR 1.12/2.7 ms, matrix size 256 × 144 and FOV 360 × 270 mm^2^. Following a bolus of contrast (total 0.2 mmol/kg, gadobutrol), post-contrast T1 maps were acquired with the same slice position as the native T1 maps. Extracellular volume (ECV) maps in three short-axis slices were generated from native T1 and post-contrast T1 maps, calibrated by the hematocrit^22,23^. Moreover, LGE images were acquired in three long-axis and short-axis slices post-contrast using a free breathing phase-sensitive inversion recovery (PSIR) sequence with segmented fast low angle shot (FLASH) read out. Imaging parameters were image matrix 256 × 156, voxel size 1.3 × 1.3 × 7 mm^3^, slice thickness 8 mm, FOV 340 × 276 mm, TR 8.25 ms, TE 3.4 ms and FA 50°.

Using a T2-prepared sequence (Siemens MyoMaps product sequence), native T2 maps were acquired in three short-axis slices. Typical imaging parameters were FA 70°, pixel size 1.4 × 1.4 mm^2^, slice thickness 8.0 mm, acquisition window 700 ms, TE/TR 1.06/2.49 ms, matrix size 144 × 256 mm^2^.

### Image analysis

All images were anonymized and analyzed offline with the freely available software Segment (version 2.7 Medviso AB, Lund, Sweden)^24,25^. Left ventricle (LV) mass and volumes were quantified by delineating the endo- and epicardial borders in end-diastole and end-systole in the cine short-axis stack. Native T1, native T2, ECV and perfusion maps were analyzed by careful manual delineation of the endo- and epicardial borders of the LV in the respective short-axis stack and values were acquired in a 16-segment model of the LV^26^. To avoid blood pool and adjacent tissues contaminating the analysis, a 10% erosion margin was set for both endo- and epicardial borders. Inter-observer variability was assessed in all 30 study-participants by two independent observers and intra-observer variability was assessed by one observer re-analyzing 10 subjects, with regards to native T1, native T2, ECV and stress and rest myocardial perfusion maps.

### Statistical analysis

Continuous data were presented as median [interquartile range] or mean ± standard deviation (SD) and categorical data were presented as numbers (percentages). The Shapiro–Wilk test was used to assess normality. LV mass and volumes were indexed to body surface area (BSA), which was calculated using the Mosteller formula^27^. Global native T1, native T2, ECV, and rest and stress perfusion were acquired by averaging segmental values per subject. The RPP was calculated as heart rate multiplied by systolic blood pressure. The MPR was calculated as stress perfusion divided by rest perfusion. CMR findings between baseline, 6 months follow-up and long-term follow-up were compared using the paired t-test. Continuous variables were compared between the MINOCA patients and the volunteers with the independent t-test in normally distributed data and the Mann–Whitney U test in non-normally distributed data. Categorical data were compared between the MINOCA patients and the volunteers with Fisher’s exact test. Subgroup analysis was performed using the independent t-test or the Mann–Whitney U test. Perfusion and MPR were compared in MINOCA patients and volunteers without hypertension, and pooled MINOCA patients and volunteers were compared based on sex, presence of hypertension or statin treatment. Inter- and intra-observer agreement was calculated for global native T1, native T2, ECV and perfusion in rest and stress as well as MPR, and presented as intra-class correlation coefficient (ICC). ICC was 0.92–1.00 (*p* < 0.001 for all). Based on previously published data on healthy subjects, an a priori power calculation showed that 16 subjects were needed to detect a 0.78 ml/min/g difference in stress perfusion^28^ with 80% power and alpha of 0.05. Statistical analysis was performed with Microsoft Excel (Microsoft, Redmond, Washington, USA) and IBM SPSS Statistics (IBM SPSS Statistics version 28, IBM, New York, USA). In all statistical analyses, the significance level was defined as *p* < 0.05.

### Ethics approval and consent to participate

The study has been approved by the Swedish Ethical Review Authority, DNR 2014/131-31/1, 2017/2415-32/1, 2021-06837-02, and all patients provided written informed consent.

## Results

### Clinical characteristics and CMR findings

Clinical characteristics and CMR findings of the MINOCA patients at baseline, 6 months follow-up and long-term follow-up are presented in Table 1. There were no differences in LV volumes or mass, except for a slight increase in LV end-systolic volume between index and 6 months follow-up. Subendocardial LGE was present in one MINOCA patient that had an symptomatic MI during the initial 6 months follow-up period^19^, and the infarcted segments were excluded from analysis at long-term follow-up. The other MINOCA patients suffered no major adverse cardiac event (MACE) during follow-up.

**Table 1:** Clinical characteristics and CMR findings of MINOCA patients.

Clinical characteristics and CMR findings	Index	6 months follow-up*	*p*	Long term follow-up	*p*
Imaging from event	3 [3–4] days	190 [186–208] days		5 [3–6] years	
Age (years)	54 ± 8	55 ± 8	0.27	59 ± 7	** < 0.001**
Height (cm)	172 ± 11	173 ± 9	0.09	173 ± 10	0.37
Weight (kg)	74 ± 14	77 ± 10	0.22	75 ± 15	0.51
BSA (m^2^)	1.9 ± 0.2	1.9 ± 0.2	0.17	1.9 ± 0.2	0.45
LVM (g)	119 ± 32	130 ± 30	0.06	119 ± 29	0.12
LVMi (g/m^2^)	63 ± 12	67 ± 13	0.23	63 ± 12	0.20
LVEDV (ml)	151 ± 36	155 ± 32	0.32	151 ± 35	0.40
LVEDVi (ml/m^2^)	80 ± 13	81 ± 14	0.93	79 ± 15	0.85
LVESV (ml)	65 ± 18	67 ± 18	**0.04**	66 ± 20	0.85
LVESVi (ml/m^2^)	35 ± 8	35 ± 9	0.23	35 ± 10	0.78
LVSV (ml)	88 ± 19	89 ± 17	0.81	85 ± 17	0.26
LVSVi (ml/m^2^)	47 ± 7	46 ± 7	0.39	45 ± 7	0.58
LVEF (%)	59 ± 6	57 ± 6	0.26	57 ± 6	0.36
TnT (ng/L) at CMR	28 [15–58]				
Maximal TnT (ng/L)	82 [37–112]				
Nt-proBNP (ng/L)	111 [60–186]				

Days between index and CMR scans as well as biochemistry at baseline are presented as median [interquartile range]. CMR findings are presented as mean ± standard deviation. P-values denotes the paired t-test. *Data missing for two MINOCA patients, i signifies indexed to body surface area (BSA) according to the Mosteller formula. Significant values are in bold.

*CMR* cardiovascular magnetic resonance imaging, *MINOCA* myocardial infarction with nonobstructive coronary arteries, *NT-proBNP* N-terminal pro–brain natriuretic peptide, *LVM* left ventricular mass, *LVEDV* left ventricular end-diastolic volume, *LVESV* left ventricular end-systolic volume, *LVSV* left ventricular stroke volume, *LVEF* left ventricular ejection fraction, *TnT* troponin T.

Clinical characteristics including comorbidities and medications of the MINOCA patients at long-term follow-up and the age- and sex-matched volunteers without IHD are presented in Table 2. The MINOCA patients were more frequently treated with statins than the volunteers, otherwise there were no differences in clinical characteristics or SAQ-7 summary score between the groups. Table 3 summarizes the CMR findings of the two groups. T2 maps could not be acquired in one MINOCA patient due to operator dependency. There were no differences in hemodynamic parameters, LV volumes or mass, native T1, native T2 or ECV.

**Table 2:** Clinical characteristics of MINOCA patients and volunteers.

Clinical characteristics	MINOCA (n = 15)	Volunteers (n = 15)	*p*
Female sex, n (%)	9 (60%)	9 (60%)	1.00
Age (years)	59 ± 7	59 ± 7	0.85
Height (cm)	173 ± 10	173 ± 10	0.94
Weight (kg)	75 ± 15	71 ± 14	0.40
BSA(m^2^)	1.9 ± 0.2	1.8 ± 0.2	0.47
Creatinine (mmol/l)	71 ± 17	74 ± 12	0.40
EVF (%)	43 ± 5	43 ± 4	0.92
Hypertension, n (%)	8 (57%)	3 (20%)	0.06
Diabetes mellitus, n (%)	2 (14%)	1 (7%)	0.60
Hyperlipidemia, n (%)	5 (36%)	2 (13%)	0.39
Smoking currently, n (%)	4 (29%)	1 (7%)	0.17
Smoking previously, n (%)	9 (64%)	6 (40%)	0.14
Statins, n (%)	7 (50%)	1 (7%)	**0.04**
Beta blockers, n (%)	3 (21%)	2 (13%)	0.65
ACE-I/ARB, n (%)	6 (43%)	2 (13%)	0.11
Calcium channel blockers, n (%)	2 (14%)	1 (7%)	0.60
SAQ-7 summary score	100 [97–100]*	100 [100–100]	0.16^†^

Clinical characteristics are presented as n (%) where p-values denotes the Fisher’s exact test or as median [interquartile range] or mean ± standard deviation where p-values denotes the independent t-test and † denotes the Mann Whitney U test. *Data missing for one patient. Significant values are in bold.

*MINOCA* myocardial infarction with nonobstructive coronary arteries, *BSA* body surface area, *EVF* erythrocyte volume fraction, *ACE-I* angiotensin-converting-enzyme inhibitors, *ARB* angiotensin receptor blockers, *SAQ-7* Seattle Angina Questionnaire-7.

**Table 3:** CMR findings of MINOCA patients and volunteers.

CMR findings	MINOCA (n = 15)	Volunteers (n = 15)	*p*
Heart rate rest (bpm)	67 ± 9	71 ± 11	0.21
SBP rest (mmHg)	134 ± 15	128 ± 15	0.59
RPP rest	9014 ± 1672	9114 ± 1781	0.52
Heart rate stress (bpm)	88 ± 10	90 ± 8	0.67
SBP stress (mmHg)	137 ± 18	128 ± 15	0.22
RPP stress	12083 ± 2265	11506 ± 1699	0.52
CO (l/min)	5.1 ± 0.8	5.7 ± 1.3	0.30
LVM (ml)	114 ± 27	102 ± 25	0.29
LVM (g)	119 ± 29	107 ± 27	0.29
LVMi (g/m^2^)	63 ± 12	58 ± 10	0.39^†^
LVEDV (ml)	151 ± 35	153 ± 41	0.98
LVEDVi (ml/m^2^)	79 ± 15	82 ± 16	0.8^†^
LVESV (ml)	66 ± 20	69 ± 22	0.68
LVESVi (ml/m^2^)	35 ± 10	37 ± 9	0.49
LVSV (ml)	85 ± 17	84 ± 23	0.68
LVSVi (ml/m^2^)	45 ± 7	45 ± 9	0.87^†^
LVEF (%)	57 ± 6	55 ± 6	0.26
T1 (ms)	976 ± 55	983 ± 26	0.87^†^
T2 (ms)	49 ± 3*	49 ± 3	0.92
ECV (%)	27 ± 3	27 ± 2	0.69
Perfusion rest (ml/min/g)	0.96 ± 0.19	1.12 ± 0.33*	0.18
Perfusion stress (ml/min/g)	2.83 ± 1.84	3.53 ± 0.73	**0.02**
MPR	3 ± 0.9	3.2 ± 1*	0.43

CMR findings are presented as mean ± standard deviation. P-value denotes the independent t-test and p-values marked † denotes the Mann Whitney U test. *T2 maps missing for one MINOCA patient, rest perfusion maps and MPR missing for one healthy volunteer, i signifies indexed to body surface area (BSA) according to the Mosteller formula. Significant values are in bold.

*CMR* cardiovascular magnetic resonance imaging, *MINOCA* myocardial infarction with nonobstructive coronary arteries, *bpm* beats per minute, *SBP* systolic blood pressure, *RPP* rate pressure product, *CO* cardiac output, *LVM* left ventricular mass, *LVEDV* left ventricular end-diastolic volume, *LVESV* left ventricular end-systolic volume, *LVSV* left ventricular stroke volume, *LVEF* left ventricular ejection fraction, *bpm* beats per minute, *ECV* extracellular volume, *MPR* myocardial perfusion reserve.

### Perfusion in stress and rest

**Figure 2:**
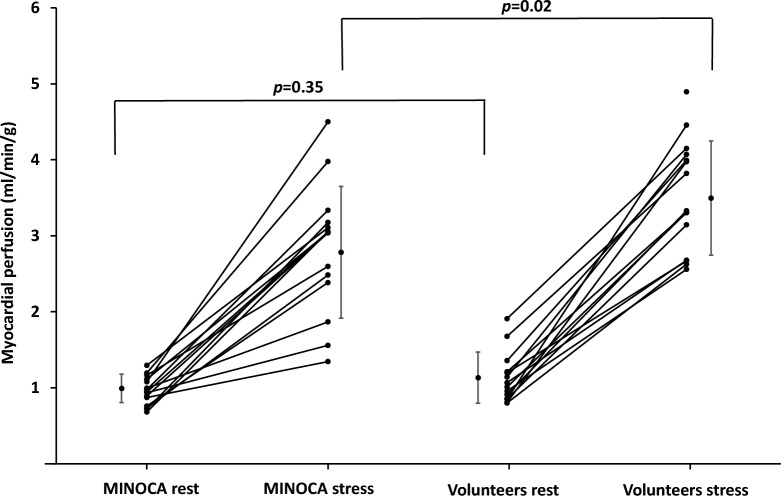
Rest and stress perfusion in MINOCA patients and volunteers. The figure shows individual global rest and stress perfusion (ml/min/g) in MINOCA patients (n = 15) and volunteers (n = 15, however rest perfusion missing in one volunteer). Stress perfusion is reduced in the MINOCA group, while rest perfusion does not differ between the groups. P-values denotes the independent t-test, error bars represent mean and standard deviation. *MINOCA* myocardial infarction with nonobstructive coronary arteries.

**Figure 3:**
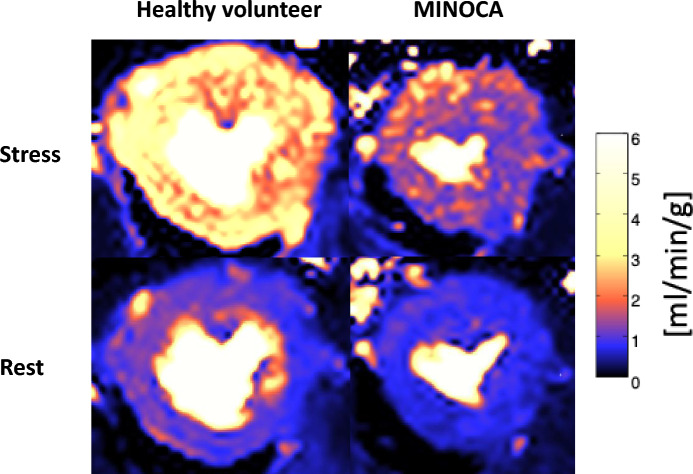
Stress and rest perfusion in a healthy volunteer and a MINOCA patient with CMD. The figure shows a representative example of midventricular perfusion maps (ml/min/g) in stress and rest in a volunteer without ischemic heart disease and a patient with MINOCA and CMD. Stress perfusion is globally reduced in the MINOCA patient compared to the volunteer, while rest perfusion is comparable. *MINOCA* myocardial infarction with nonobstructive coronary arteries, *CMD* coronary microvascular dysfunction.

The MINOCA patients had lower global stress perfusion compared to volunteers (2.83 ± 1.84 vs 3.53 ± 0.73, *p* = 0.02), however there were no differences in rest perfusion or MPR, as shown in Table 3. Figure 2 shows individual rest and stress perfusion values of the MINOCA patients and the volunteers without symptomatic IHD with mean and standard deviation between the groups, illustrating that MINOCA patients have lower stress perfusion. Rest perfusion maps were excluded in one volunteer due to residual hyperemia. Representative examples of midventricular perfusion maps in stress and rest of a MINOCA patient with suspected CMD and a healthy volunteer are shown in Fig. 3. Stress perfusion was lower in MINOCA patients without hypertension compared to volunteers without hypertension (2.71 ± 0.96 vs 3.64 ± 0.75 ml/min/g, *p* = 0.04), however there were no differences in rest perfusion or MPR (*p* > 0.05, data not shown). There were no differences in rest or stress perfusion, or MPR, between pooled MINOCA patients and volunteers compared with regards to sex or presence of hypertension or statin treatment (*p* > 0.05, data not shown).

## Discussion

To the best of our knowledge, this is the first study to assess presence of CMD during long-term follow-up of MINOCA patients with an initial normal CMR scan, using fully quantitative stress CMR perfusion mapping. The MINOCA patients had lower stress perfusion compared to age- and sex-matched volunteers with similar cardiovascular risk factors but without IHD, suggesting presence of CMD. However, it is unknown if CMD was present before and at hospitalization at index event or may have developed during follow-up due to increasing age or burden of cardiovascular risk factors.

### Coronary microvascular dysfunction in MINOCA

Early CMR is recommended for differential diagnosis in MINOCA and provides a diagnosis in most cases, which carry prognostic importance with regards to future risk of MACE and potential specific treatment^29^. Specifically, presence of LGE and abnormal T2 mapping values have been shown to be high-risk markers in follow-up of MINOCA patients^30^. Despite early comprehensive CMR including examination of presence of myocardial edema and injury using T1/T2 mapping, LGE and ECV mapping, more than 25% of MINOCA cases have normal CMR scans^29^. However, further examinations of pathologies not detectable on traditional CMR is warranted. The role of CMD in MINOCA is increasingly recognized but cannot be diagnosed using traditional CMR^18^. Indeed, the workup of CMD is complex due to the many pathophysiological forms of CMD that patients present with and includes various modalities^18^. Microvascular spasm has been shown in acute MINOCA using intracoronary provocative testing immediately following diagnostic CAG^31–33^, which was associated with increased risk of MACE during 3 years follow-up^31^. Increased microvascular constrictor reactivity and impaired microvascular dilatation has been shown in MINOCA at index and 12-months follow-up by evaluating blood flow response to ergonovine, adenosine and cold pressor test with transthoracic Doppler echocardiography^34^. Moreover, increased CAG-derived index of microcirculatory resistance (IMR) has been shown in acute MINOCA, which was associated with increased risk of MACE during 2 years follow-up^5^. Interestingly, increased CAG-derived IMR has recently been shown across different subtypes of MINOCA, indicating CMD to be a unifying pathophysiological feature of several different clinical entities^35^. The only prior CMR study evaluating CMD in acute MINOCA, using qualitative perfusion analysis, showed focal rest and stress perfusion abnormalities, which were often co-localized with T2 + and/or ischemic or non-ischemic LGE^4^. MPR index (MPRI) was low in areas both with and without LGE and T2 + ^4^. Thus, it is unclear if ischemia caused LGE and T2+, or if acute edema caused LGE and T2 + as well as hypoperfusion by compressing micro-vessels^4^. This contrasts with the present follow-up study, where the only difference was reduced stress perfusion. Since native T1, native T2 and ECV did not differ between MINOCA patients and volunteers, the reduced stress perfusion cannot be attributed to edema or fibrosis from previous myocarditis or MI. One MINOCA patient in our study had LGE from a previous MI, occurring during initial 6 months follow-up^19^ and affected segments were excluded from analysis. The MINOCA patients had a nonobstructive CAG at presentation, and no patient had focal perfusion defects on stress perfusion CMR. Therefore, MI or obstructive CAD cannot explain reduced stress perfusion in the MINOCA patients. As the MINOCA patients had no other pathophysiological explanation detectable at index and were compared to age- and sex-matched volunteers with similar cardiovascular risk factors but without a history of IHD, we theorize that their initial presentation may be attributable to CMD which remained at follow-up.

### Coronary microvascular dysfunction and quantitative CMR perfusion mapping

Novel fully quantitative adenosine stress CMR perfusion mapping adds the possibility to identify CMD, presenting as reduced global stress perfusion and/or MPR, which has been shown in patients with post-covid^36^, heart failure with preserved ejection fraction (HFpEF)^37^, dilated cardiomyopathy (DCM)^38^, and microvascular angina pectoris (without obstructive CAD)^17,39–41^. Moreover, quantitative CMR perfusion mapping can detect obstructive CAD^42^ and differentiate CMD from three-vessel CAD which causes more pronounced reduction in myocardial perfusion without focal perfusion defects^17^.

CMD is common in HFpEF, as shown by reduced MPR despite unaffected stress perfusion due to increased rest perfusion, and ECV is increased^37^. Stress perfusion and MPR is reduced in DCM while rest perfusion is augmented, with lower rest and stress perfusion in segments with LGE^38^. The increased rest perfusion in both studies may indicate functional CMD, as reduced resting tone and higher rest perfusion has been shown invasively in functional CMD, causing reduced stress perfusion due to impaired vasodilatory reserve^43^. However, presence of fibrosis indicates structural CMD, possibly coexisting and contribution to each other. In microvascular angina, stress perfusion and MPR is reduced while rest perfusion is unaffected^17,39^. Moreover, reduced stress perfusion and MPR, without differences in T1, ECV or rest perfusion, has been shown in microvascular angina^40^. These studies report lower stress perfusion and MPR than the current study, possibly reflecting more pronounced CMD also contributing to clinical ischemic symptoms. Moreover, women have higher perfusion values, which decreases with age^28,44^. Two studies included more men of higher age^17,39^ and one included more females of similar age compared to the current study^40^. Furthermore, the results of these studies may reflect structural CMD, as normal rest perfusion and impaired stress perfusion has been shown invasively in structural CMD, due to impaired vasodilatation and/or increased microvascular resistance^43^. Overall, the compared studies show the complexity of CMD as common pathophysiological feature in many different clinical entities, with many different mechanisms and presentations ultimately impairing myocardial perfusion.

## Strengths and limitations

Fully quantitative stress CMR perfusion mapping avoids risks and limitations related to CAG and positron emission tomography (PET), the current gold-standard modalities for assessing CMD^15,17,39^. The MINOCA patient group was well-characterized, presenting with an MI with nonobstructive coronary arteries as determined visually on CAG and a normal CMR scan at index hospitalization and 6 months follow-up. One individual had an MI during initial 6 months-follow-up^19^, and the infarcted segments were excluded from analysis at long-term follow-up. Optical coherence tomography or intravascular ultrasound was not performed at index hospitalization and therefore, plaque erosion or rupture could have been overlooked. Moreover, it is unknown if CMD was present before and at index hospitalization or developed during follow-up. However, the MINOCA patients were compared to volunteers with no history of IHD despite matching age and sex, and similar cardiovascular risk factors. The MINOCA patients were more frequently treated with statins and had a numerically higher prevalence of hypertension. Hypertension and hyperlipidemia are known risk factors for CMD, decreasing stress perfusion and/or MPRI^45–49^. The small sample size in the current study does indeed limit the utility of subgroup analysis. However, also normotensive MINOCA patients had lower stress perfusion than normotensive volunteers and there were no differences comparing pooled MINOCA patients and volunteers based on hypertension or statin use, with regards to stress and rest perfusion as well as MPR. Intra- and interobserver agreement was excellent, indicating that quantitative perfusion mapping is a robust method for evaluating myocardial perfusion. The loss to follow-up during this long-term study with the resultant small sample size constitutes major limitations. Sample sizes were larger in the compared studies^17,36–40^, however the sample size calculation prior to study start established 16 patients as sufficient based on previous stress perfusion values. Unfortunately, T2 maps were missing in one MINOCA patient and rest perfusion maps of one healthy volunteer were excluded due to residual hyperemia. Neither T2 values nor rest perfusion were the primary scope of this study, thus the effect of these missing values is small, as there is no missing data for stress perfusion which was the primary aim of this study. Furthermore, perfusion values of the volunteers were similar to previously reported normal values^14,28,40,50^, thus possibly reflecting healthy perfusion values. Further multi-center studies in larger groups both at admission and during long-term follow-up are needed to further elucidate the role of CMD in MINOCA, and the results of this study may be considered as hypothesis generating for future studies to come.

## Conclusions

Global stress perfusion is lower in MINOCA patients with a normal initial CMR scan, during long-term follow-up, compared to age- and sex-matched volunteers with similar cardiovascular risk factors but without a history of IHD. Therefore, it is possible that CMD may be a pathophysiological mechanism in MINOCA.

## Data Availability

Data supporting the findings in this study are available from the corresponding author upon reasonable request.

## Abbreviations

CAD: Coronary artery disease
CMD: Coronary microvascular dysfunction
CMR: Cardiovascular magnetic resonance imaging
ECV: Extracellular volume
IHD: Ischemic heart disease
MI: Myocardial infarction
MINOCA: Myocardial infarction with nonobstructive coronary arteries
MPR: Myocardial perfusion reserve

## References

[1] Thygesen K, Alpert JS, Jaffe AS, Chaitman BR, Bax JJ, Morrow DA (2018). Fourth universal definition of myocardial infarction (2018). Circulation..

[2] Tamis-Holland JE, Jneid H, Reynolds HR, Agewall S, Brilakis ES, Brown TM (2019). Contemporary diagnosis and management of patients with myocardial infarction in the absence of obstructive coronary artery disease: A scientific statement from the American heart association. Circulation..

[3] Collet JP, Thiele H, Barbato E, Barthélémy O, Bauersachs J, Bhatt DL (2021). 2020 ESC Guidelines for the management of acute coronary syndromes in patients presenting without persistent ST-segment elevation. Eur. Heart J..

[4] Mauricio R, Srichai MB, Axel L, Hochman JS, Reynolds HR (2016). Stress cardiac MRI in women with myocardial infarction and nonobstructive coronary artery disease. Clin. Cardiol..

[5] Abdu FA, Liu L, Mohammed AQ, Yin G, Xu B, Zhang W (2021). Prognostic impact of coronary microvascular dysfunction in patients with myocardial infarction with non-obstructive coronary arteries. Eur. J. Intern. Med..

[6] Vancheri F, Longo G, Vancheri S, Henein M (2020). Coronary microvascular dysfunction. J. Clin. Med..

[7] Montone RA, Jang IK, Beltrame JF, Sicari R, Meucci MC, Bode M (2021). The evolving role of cardiac imaging in patients with myocardial infarction and non-obstructive coronary arteries. Prog. Cardiovasc. Dis..

[8] Reynolds HR (2022). Should every patient with MINOCA have cardiac magnetic resonance?. JACC Cardiovasc. Imaging..

[9] Agewall S, Beltrame JF, Reynolds HR, Niessner A, Rosano G, Caforio AL (2017). ESC working group position paper on myocardial infarction with non-obstructive coronary arteries. Eur. Heart J..

[10] Liang K, Nakou E, Del Buono MG, Montone RA, D'Amario D, Bucciarelli-Ducci C (2021). The role of cardiac magnetic resonance in myocardial infarction and non-obstructive coronary arteries. Front. Cardiovasc. Med..

[11] Emrich T, Kros M, Schoepf UJ, Geyer M, Mildenberger P, Kloeckner R (2021). Cardiac magnetic resonance imaging features prognostic information in patients with suspected myocardial infarction with non-obstructed coronary arteries. Int. J. Cardiol..

[12] Machanahalli Balakrishna A, Ismayl M, Thandra A, Walters R, Ganesan V, Anugula D (2022). Diagnostic value of cardiac magnetic resonance imaging and intracoronary optical coherence tomography in patients with a working diagnosis of myocardial infarction with non-obstructive coronary arteries—a systematic review and meta-analysis. Curr. Probl. Cardiol..

[13] Sörensson P, Ekenbäck C, Lundin M, Agewall S, Bacsovics Brolin E, Caidahl K (2021). Early comprehensive cardiovascular magnetic resonance imaging in patients with myocardial infarction with nonobstructive coronary arteries. JACC Cardiovasc. Imaging..

[14] Kellman P, Hansen MS, Nielles-Vallespin S, Nickander J, Themudo R, Ugander M (2017). Myocardial perfusion cardiovascular magnetic resonance: Optimized dual sequence and reconstruction for quantification. J. Cardiovasc. Magn. Reson..

[15] Engblom H, Xue H, Akil S, Carlsson M, Hindorf C, Oddstig J (2017). Fully quantitative cardiovascular magnetic resonance myocardial perfusion ready for clinical use: A comparison between cardiovascular magnetic resonance imaging and positron emission tomography. J. Cardiovasc. Magn. Reson..

[16] Hamilton-Craig C, Ugander M, Greenwood JP, Kozor R (2022). Stress perfusion cardiovascular magnetic resonance imaging: A guide for the general cardiologist. Heart..

[17] Kotecha T, Martinez-Naharro A, Boldrini M, Knight D, Hawkins P, Kalra S (2019). Automated pixel-wise quantitative myocardial perfusion mapping by CMR to detect obstructive coronary artery disease and coronary microvascular dysfunction: Validation against invasive coronary physiology. JACC Cardiovasc. Imaging..

[18] Mohammed AQ, Abdu FA, Liu L, Yin G, Mareai RM, Mohammed AA (2023). Coronary microvascular dysfunction and myocardial infarction with non-obstructive coronary arteries: Where do we stand?. Eur. J. Intern. Med..

[19] Nickander J, Ekenbäck C, Agewall S, Brolin EB, Caidahl K, Cederlund K (2023). Comprehensive follow-up cardiac magnetic resonance of patients with myocardial infarction with nonobstructive coronary arteries. JACC Cardiovasc. Imaging.

[20] Chan PS, Jones PG, Arnold SA, Spertus JA (2014). Development and validation of a short version of the Seattle angina questionnaire. Circ. Cardiovasc. Qual. Outcomes..

[21] Bassingthwaighte JB, Wang CY, Chan IS (1989). Blood-tissue exchange via transport and transformation by capillary endothelial cells. Circ. Res..

[22] Kellman P, Wilson JR, Xue H, Ugander M, Arai AE (2012). Extracellular volume fraction mapping in the myocardium, part 1: evaluation of an automated method. J. Cardiovasc. Magn. Reson..

[23] Arheden H, Saeed M, Higgins CB, Gao DW, Bremerich J, Wyttenbach R (1999). Measurement of the distribution volume of gadopentetate dimeglumine at echo-planar MR imaging to quantify myocardial infarction: Comparison with 99mTc-DTPA autoradiography in rats. Radiology..

[24] Heiberg E, Sjögren J, Ugander M, Carlsson M, Engblom H, Arheden H (2010). Design and validation of segment-freely available software for cardiovascular image analysis. BMC Med. Imaging..

[25] Tufvesson J, Hedström E, Steding-Ehrenborg K, Carlsson M, Arheden H, Heiberg E (2015). Validation and development of a new automatic algorithm for time-resolved segmentation of the left ventricle in magnetic resonance imaging. Biomed. Res. Int..

[26] Hundley WG, Bluemke D, Bogaert JG, Friedrich MG, Higgins CB, Lawson MA (2009). Society for cardiovascular magnetic resonance guidelines for reporting cardiovascular magnetic resonance examinations. J. Cardiovasc. Magn. Reson..

[27] Mosteller RD (1987). Simplified calculation of body-surface area. N. Engl. J. Med..

[28] Nickander J, Themudo R, Sigfridsson A, Xue H, Kellman P, Ugander M (2020). Females have higher myocardial perfusion, blood volume and extracellular volume compared to males—an adenosine stress cardiovascular magnetic resonance study. Sci. Rep..

[29] Mileva N, Paolisso P, Gallinoro E, Fabbricatore D, Munhoz D, Bergamaschi L (2023). Diagnostic and prognostic role of cardiac magnetic resonance in MINOCA: Systematic review and meta-analysis. JACC Cardiovasc. Imaging..

[30] Bergamaschi L, Foà A, Paolisso P, Renzulli M, Angeli F, Fabrizio M (2023). Prognostic role of early cardiac magnetic resonance in myocardial infarction with nonobstructive coronary arteries. JACC Cardiovasc. Imaging..

[31] Montone RA, Niccoli G, Fracassi F, Russo M, Gurgoglione F, Cammà G (2018). Patients with acute myocardial infarction and non-obstructive coronary arteries: Safety and prognostic relevance of invasive coronary provocative tests. Eur. Heart J..

[32] Montone RA, Niccoli G, Russo M, Giaccari M, Del Buono MG, Meucci MC (2020). Clinical, angiographic and echocardiographic correlates of epicardial and microvascular spasm in patients with myocardial ischaemia and non-obstructive coronary arteries. Clin. Res. Cardiol..

[33] Pirozzolo G, Seitz A, Athanasiadis A, Bekeredjian R, Sechtem U, Ong P (2020). Microvascular spasm in non-ST-segment elevation myocardial infarction without culprit lesion (MINOCA). Clin. Res. Cardiol..

[34] De Vita A, Manfredonia L, Lamendola P, Villano A, Ravenna SE, Bisignani A (2019). Coronary microvascular dysfunction in patients with acute coronary syndrome and no obstructive coronary artery disease. Clin. Res. Cardiol..

[35] Milzi A, Dettori R, Lubberich RK, Reith S, Frick M, Burgmaier K (2023). Coronary microvascular dysfunction is a hallmark of all subtypes of MINOCA. Clin. Res. Cardiol..

[36] Doeblin P, Steinbeis F, Scannell CM, Goetze C, Al-Tabatabaee S, Erley J (2022). Brief research report: quantitative analysis of potential coronary microvascular disease in suspected long-COVID syndrome. Front. Cardiovasc. Med..

[37] Löffler AI, Pan JA, Balfour PC, Shaw PW, Yang Y, Nasir M (2019). Frequency of coronary microvascular dysfunction and diffuse myocardial fibrosis (measured by cardiovascular magnetic resonance) in patients with heart failure and preserved left ventricular ejection fraction. Am. J. Cardiol..

[38] Gulati A, Ismail TF, Ali A, Hsu LY, Gonçalves C, Ismail NA (2019). Microvascular dysfunction in dilated cardiomyopathy: A quantitative stress perfusion cardiovascular magnetic resonance study. JACC Cardiovasc. Imaging..

[39] Knott KD, Camaioni C, Ramasamy A, Augusto JA, Bhuva AN, Xue H (2019). Quantitative myocardial perfusion in coronary artery disease: A perfusion mapping study. J. Magn. Reson. Imaging..

[40] Zorach B, Shaw PW, Bourque J, Kuruvilla S, Balfour PC, Yang Y (2018). Quantitative cardiovascular magnetic resonance perfusion imaging identifies reduced flow reserve in microvascular coronary artery disease. J. Cardiovasc. Magn. Reson..

[41] Rahman H, Scannell CM, Demir OM, Ryan M, McConkey H, Ellis H (2021). High-resolution cardiac magnetic resonance imaging techniques for the identification of coronary microvascular dysfunction. JACC Cardiovasc. Imaging..

[42] Zhao SH, Guo WF, Yao ZF, Yang S, Yun H, Chen YY (2023). Fully automated pixel-wise quantitative CMR-myocardial perfusion with CMR-coronary angiography to detect hemodynamically significant coronary artery disease. Eur. Radiol..

[43] Sinha A, Rahman H, Perera D (2020). Ischaemia without obstructive coronary artery disease: The pathophysiology of microvascular dysfunction. Curr. Opin. Cardiol..

[44] Brown LAE, Gulsin GS, Onciul SC, Broadbent DA, Yeo JL, Wood AL (2022). Sex- and age-specific normal values for automated quantitative pixel-wise myocardial perfusion cardiovascular magnetic resonance. Eur. Heart J. Cardiovasc. Imaging..

[45] Wang L, Jerosch-Herold M, Jacobs DR, Shahar E, Folsom AR (2006). Coronary risk factors and myocardial perfusion in asymptomatic adults: The Multi-Ethnic Study of Atherosclerosis (MESA). J. Am. Coll. Cardiol..

[46] Nakajima H, Onishi K, Kurita T, Ishida M, Nagata M, Kitagawa K (2010). Hypertension impairs myocardial blood perfusion reserve in subjects without regional myocardial ischemia. Hypertens Res..

[47] Cha MJ, Kim SM, Kim HS, Kim Y, Choe YH (2018). Association of cardiovascular risk factors on myocardial perfusion and fibrosis in asymptomatic individuals: cardiac magnetic resonance study. Acta Radiol..

[48] Zhou W, Lee JCY, Leung ST, Lai A, Lee TF, Chiang JB (2021). Long-term prognosis of patients with coronary microvascular disease using stress perfusion cardiac magnetic resonance. JACC Cardiovasc. Imaging..

[49] Kawecka-Jaszcz K, Czarnecka D, Olszanecka A, Klecha A, Kwiecień-Sobstel A, Stolarz-Skrzypek K (2008). Myocardial perfusion in hypertensive patients with normal coronary angiograms. J. Hypertens..

[50] Hsu LY, Jacobs M, Benovoy M, Ta AD, Conn HM, Winkler S (2018). Diagnostic performance of fully automated pixel-wise quantitative myocardial perfusion imaging by cardiovascular magnetic resonance. JACC Cardiovasc. Imaging..

